# Inflammatory Immune Responses in Patients with Tick-Borne Encephalitis: Dynamics and Association with the Outcome of the Disease

**DOI:** 10.3390/microorganisms7110514

**Published:** 2019-10-31

**Authors:** Petra Bogovič, Lara Lusa, Miša Korva, Stanka Lotrič-Furlan, Katarina Resman-Rus, Miša Pavletič, Tatjana Avšič-Županc, Klemen Strle, Franc Strle

**Affiliations:** 1 Department of Infectious Diseases, University Medical Center Ljubljana, Japljeva 2, 1525 Ljubljana, Slovenia; stanka.lotric-furlan@mf.uni-lj.si (S.L.-F.); franc.strle@kclj.si (F.S.); 2 Faculty of Medicine, University of Ljubljana, Vrazov trg 2, 1000 Ljubljana, Slovenia; 3 Institute for Biostatistics and Medical Informatics, Faculty of Medicine, University of Ljubljana, Vrazov trg 2, 1000 Ljubljana, Slovenia; lara.lusa@mf.uni-lj.si; 4 Department of Mathematics, Faculty of Mathematics, Natural Sciences and Information Technologies, University of Primorska, Glagoljaška 8, 6000 Koper, Slovenia; 5 Institute of Microbiology and Immunology, Faculty of Medicine, University of Ljubljana, Zaloška 4, 1000 Ljubljana, Slovenia; Misa.Korva@mf.uni-lj.si (M.K.); katarina.resman@mf.uni-lj.si (K.R.-R.); Misa.Pavletic@mf.uni-lj.si (M.P.); Tatjana.Avsic@mf.uni-lj.si (T.A.-Ž.); 6 Division of Rheumatology, Allergy and Immunology, Center for Immunology and Inflammatory Diseases, Masachusetts General Hospital/Harvard Medical School, 55 Fruit Street, Boston, MA 02114, USA; KSTRLE@mgh.harvard.edu

**Keywords:** tick-borne encephalitis, outcome, post-encephalitic syndrome, inflammatory mediators, cytokines, chemokines, immunopathogenesis, innate immune responses, adaptive immune responses

## Abstract

Information on the association of inflammatory immune responses and disease outcome after tick-borne encephalitis (TBE) is limited. In the present study, we assessed the levels of 24 cytokines/chemokines associated with innate and adaptive immune responses in matched serum and cerebrospinal fluid (CSF) samples of 81 patients at first visit, and in serum at follow-up time points. Serum levels of several cytokines/chemokines obtained during the meningoencephalitic phase of TBE differed compared to the levels at a follow-up visit 2 months later; several significant differences were also found in cytokine/chemokine levels in serum at 2 months compared to the last time point, 2–7 years after acute illness. Cytokines/chemokines levels in CSF or serum obtained at the time of acute illness or serum levels obtained 2 months after the onset of TBE did not have predictive value for an unfavorable outcome 2–7 years later. In contrast, serum levels of mediators associated with Th17 responses were lower in patients with unfavorable outcome whereas those associated with other adaptive or innate immune responses were higher at the last visit in those with an unfavorable outcome. These findings provide new insights into the immunopathogenesis of TBE and implicate inflammatory immune responses with post-encephalitic syndrome years after the initial infection.

## 1. Introduction

Tick-borne encephalitis (TBE) is a flaviviral infection of the central nervous system, which is endemic in many parts of Europe and Asia. It is transmitted to humans predominantly through *Ixodes* spp. tick bites. There is no specific antiviral treatment for TBE, but the disease is preventable by vaccination [1,2,3,4]. TBE is caused by three subtypes of the TBE virus (TBEV); European, Siberian, and Far-Eastern. Most patients infected with the European virus subtype who develop symptoms have a biphasic disease course. The initial phase presents as mild febrile illness associated with general malaise, fatigue, headache, myalgia and arthralgia, and others. The second phase of TBE presents as meningitis in about 50% of adult patients, meningoencephalitis in 40%, or meningoencephalomyelitis in 5–10%. In children, meningitis is the predominant clinical manifestation. The case fatality rate is between 0.5 and 2%. In addition, at least 30% of patients develop a post-encephalitic syndrome (PES), and approximately 5% are affected by permanent pareses [3,4,5,6].

The pathogenesis of TBE, including the interaction of TBEV with innate and adaptive immunity is not well understood. Although the clinical presentation of TBE infection is believed to be driven predominantly by the host immune responses, knowledge of the type of immune responses involved and their impact on symptomology, severity, and outcome of TBE is limited. 

In addition to TBEV, several other members of the flavivirus genus, such as the Japanese encephalitis virus, dengue virus, and West Nile virus are pathogenic to humans and may cause encephalitis. The role played by the immune response caused by these viruses is poorly understood; more information is available on the acute illness than on the outcome. Dengue virus and Japanese encephalitis virus infection of the central nervous system induce expression of multiple cytokines, which may augment inflammation and other pathological changes in the infected brain [7]. Similar cerebrospinal fluid (CSF) levels of cytokines in dengue encephalitis and Japanese encephalitis patients were reported [7] and differences in the immunological response of the Japanese encephalitis and non-Japanese encephalitis cases were exposed [8]. It is of interest that in patients with Japanese encephalitis, dengue encephalitis, and nonspecific encephalitis, cytokines and chemokines did not correlate with severity of illness [9]. The reports on the association of the levels of inflammatory immune mediators in patients with flavivirus central nervous system infection are rare. It seems that during Japanese encephalitis virus infection, elevated levels of proinflammatory cytokines and chemokines are associated with a poor outcome, but whether they are simply a correlate of severe disease or contribute to pathogenesis remains to be determined [10]. 

In the current study, in an effort to delineate immune responses in the clinical course and outcome of TBE, we assessed 24 cytokines and chemokines associated with innate and adaptive (T and B cell) immune responses in patients with TBE who were followed systematically from the initial visit during acute illness for at least 2 years to evaluate their post-treatment status. All patients were from Central Europe (Slovenia) where the European TBEV subtype prevails. The levels of these inflammatory mediators were determined in matched CSF and serum samples at first visit, and in serum at follow-up time points, and correlated with unfavorable outcomes in TBE.

## 2. Materials and Methods

The study was approved by the Medical Ethics Committee of the Ministry of Health of the Republic of Slovenia (No 0120-467/2017/3). Each participant provided written informed consent. 

### 2.1. Evaluation of Patients

This study is based on 420 adult patients who were diagnosed with TBE in the years 2007–2012 at the Department of Infectious Diseases, UMC Ljubljana, Slovenia, and assessed for long-term outcome in 2014. Detailed clinical information and evaluation criteria on these patients, and the inflammatory profile in the acute meningoencephalitic phase of illness in a subset of them, were reported previously [11,12]. Briefly, clinical and laboratory information on the course and severity of acute illness was obtained and the outcome of TBE was assessed at follow-up visits. During hospitalization (initial visit) and at each follow-up visit, each patient was evaluated for the presence of clinical signs and subjective symptoms. Determination of TBE-associated symptoms was made if patients reported newly developed symptoms or worsening of existing symptoms since the onset of TBE without another medical explanation.

We defined TBE sequelae as objective neurological signs (ataxia, tremor, cranial and spinal nerve pareses, etc.) and subjective symptoms (headache, dizziness, concentration and memory disorders, emotional lability, sleep disorders, fatigue, myalgias and/or arthralgias, etc.) that match the criteria for TBE-associated symptoms at examination 2–7 years after the acute illness. 

An unfavorable long-term clinical outcome was defined as the existence of PES defined with the presence of two or more subjective symptoms fulfilling the criteria for TBE-associated symptoms or at least one objective neurological sign at the last follow-up visit 2–7 years after the acute illness. 

### 2.2. Selection of Patients for the Analysis of Cytokine and Chemokine Levels

The present study comprised 81 patients selected from the larger cohort of 420 patients in a previous study on the clinical assessment of long-term outcome of TBE [11,12]. Patients were selected to have comparable numbers of patients with favorable versus unfavorable outcome and based on the availability of matched CSF specimens and serum samples obtained at the time of acute illness, and serum samples at follow-up examinations at 2 months and 2–7 years after study entry. Samples were aliquoted and frozen at −80 °C for an average of 76 (48–80) months for serum and 80 (78–90) months for CSF prior to testing. Samples did not undergo freeze-thaw cycles except for this cytokine analysis. No significant differences were observed in the demographic or clinical characteristics of the 81 patients who were selected for the present study and the rest of the patients in the larger cohort, with the exception that the proportion of patients with unfavorable outcome at 2–7 years after TBE was higher in the present study due to the selection criteria. 

### 2.3. Chemokine and Cytokine Determinations

The concentration of 24 cytokines was measured in serum and CSF samples with four different Human Cytokine/Chemokine Panels (Milliplex Map, Merck, Germany) on the MagPix instrument (Luminex, Austin, TX, USA), according to the manufacturer’s instructions. Investigated cytokines/chemokines are associated with innate (GMCSF, IFNα, IL-1β, IL-6, IL-8, TNFα, MCP1 (CCL2), MIP1α (CCL3), IL-10), adaptive Th1 (IFNγ, IL-12P40, IL-12P70, CXCL10, CXCL9, CCL19), Th17 (IL-17F, IL-17A, IL-22, IL-21, IL-23, IL-25 (IL-17E), IL-27), and B cell immune response (CXCL12, CXCL13). All samples were previously aliquoted and diluted to a final concentration of 1:5. Samples in a single panel were performed on the same day and all plates were analyzed simultaneously with the Milliplex Analyst 5.1 software in order to minimize inter-assay variation. Inflammatory profiles were assessed at first visit in matched CSF and serum samples, and in serum at follow-up time points (at 2 months and 2–7 years). Samples from CSF were not available from follow-up visits.

### 2.4. Statistical Analyses

The categorical variables were summarized as frequencies and percentages (with their 95% confidence intervals (CI)), and the numerical variables as medians (interquartile ranges, IQR).

The Wilcoxon signed rank test was used to compare the levels of the 24 cytokines/chemokines in serum at subsequent follow-up times (at first visit during acute illness, 2 months later and at the last follow-up visit 2–7 years after TBE). Differences between groups were summarized as the ratio of the medians (medians at a follow-up visit to median at previous visit; values above 1 indicate greater median concentrations at the later visit). *p* values from the univariate tests and those adjusted for multiple comparisons using a multivariate permutation procedure were reported [13]. 

A similar analysis, based on the Mann-Whitney test was performed to compare the levels of the 24 cytokines/chemokines measured in the acute phase (CSF and serum), and at the two follow-up times to compare patients with PES and those with a favorable outcome of TBE at the last visit. The distributions of the mediators for patients with and without PES were displayed graphically using box and whisker plots.

Adjusted *p* values < 0.05 were considered statistically significant. Statistical analysis was performed using R statistical language (R), version 3.5.0. [14].

## 3. Results

As we reported previously, the analysis of the levels of cytokines and chemokines in matched serum and CSF samples obtained during meningoencephalitic phase of TBE revealed that cytokines and chemokines associated with innate immune responses or Th1 adaptive immune responses were significantly higher in CSF, whereas mediators associated with Th17 and B cell immune responses were generally higher in serum [12]. In the present study, we compared the levels of inflammatory mediators at first visit during acute illness and at follow-up time points to evaluate whether immune responses at each time point were associated with the outcome of the disease. Since there is no etiologic treatment for TBE, the natural course of the disease had been disrupted only by symptomatic therapy (most often analgetics, and rarely, nonsteroid antirheumatic drugs, none of our patients received steroids) in some of our patients. 

The basic clinical, laboratory, and demographic findings during the acute illness in patients included in the present study is shown in Table 1. Of 81 patients, 43 (53%) fulfilled the criteria for PES: 37 (46%) reported ≥ 2 TBE-associated symptoms, and 11 (13%) had ≥ 1 objective neurological sign at the follow-up visit 2–7 years after acute illness (five patients had objective sequellae of TBE as well as pronounced TBE-associated symptoms). 

### 3.1. Comparison of Inflammatory Responses in Serum during Acute Illness and Follow-Up

Serum samples for these analyses were available at the time of acute illness (*n* = 81, time 0), and at 2 months (*n* = 74) and 2–7 years thereafter (*n* = 81). 

The analysis of the levels of serum cytokines/chemokines at different time points identified several statistically significant differences (Table 2).

A comparison of the findings at the first visit and follow-up at 2 months revealed a statistically significant decrease for five mediators associated with innate (IFNα (*p* = 0.003), TNFα (*p* = 0.002)), adaptive Th17 (IL-21 (*p* < 0.001), IL-27 (*p* < 0.001)), and B cell (CXCL12 (*p* = 0.038)) responses. In contrast, three mediators associated with innate or Th1 adaptive responses (MCP1 (*p* < 0.001), CXCL9 (*p* = 0.023), and CCL19 (*p* < 0.001)) were significantly higher at the 2-month follow-up. It is possible that these responses may decrease during acute illness and are normalized 2 months later. 

Even greater statistically significant differences were found when comparing the cytokine and chemokine levels in serum at 2 months, and at the last time point 2–7 years later. The levels of most innate immune mediators were generally higher at the last visit, suggesting that these levels may initially be reduced 2 months after acute illness. In contrast, mediators associated with Th1 and Th17 responses generally decreased over time, whereas the levels of B cell mediators remained steady after the 2-month visit (Table 2, Figure S1).

In three patients with TBE and concomitant Lyme neuroborreliosis, the levels of cytokines/chemokines in serum and their dynamics seemed comparable to those found in patients without coinfection (data not shown).

### 3.2. Association of Cytokine and Chemokine Levels and Outcome of TBE

Since several inflammatory mediators were associated with more severe acute illness [12], we next assessed the potential association between cytokine and chemokine levels in CSF or serum at the initial visit and in serum at follow-up time points (2 months and 2–7 years thereafter) with the clinical outcome of TBE, i.e., with the presence of PES 2–7 years after TBE. 

**Figure 1:**
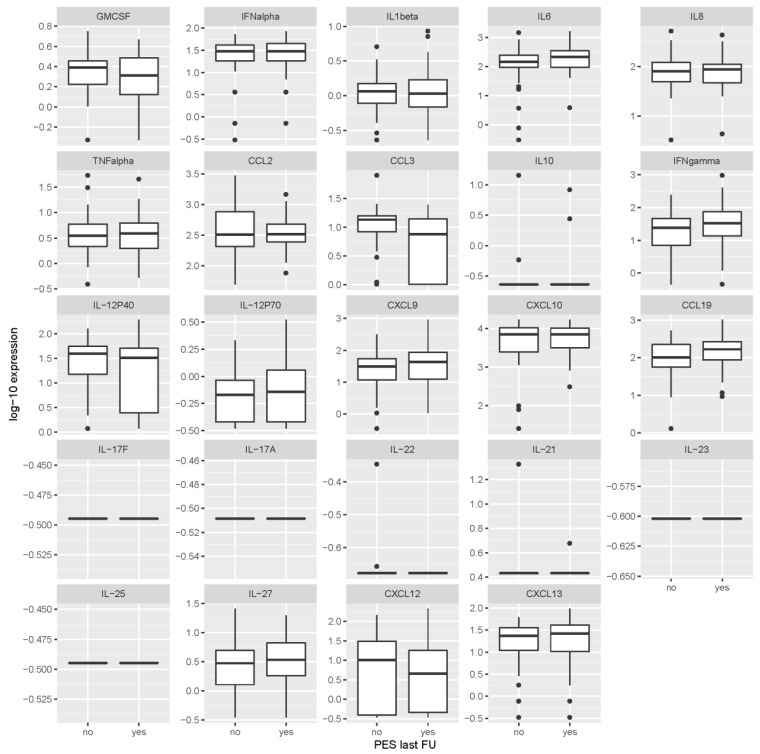
Levels of cytokines/chemokines in cerebrospinal fluid obtained during the meningoencephalitic phase of tick-borne encephalitis according to the presence or absence of post-encephalytic syndrome at the last follow-up visit (2–7 years after acute illness). Concentrations are in ng/mL for IL-17F, IL-23, IL-25 and IL-27, and in pg/mL for all other inflammatory immune mediators. PES last FU = post-encephalitic syndrome present at last follow-up visit.

**Figure 2:**
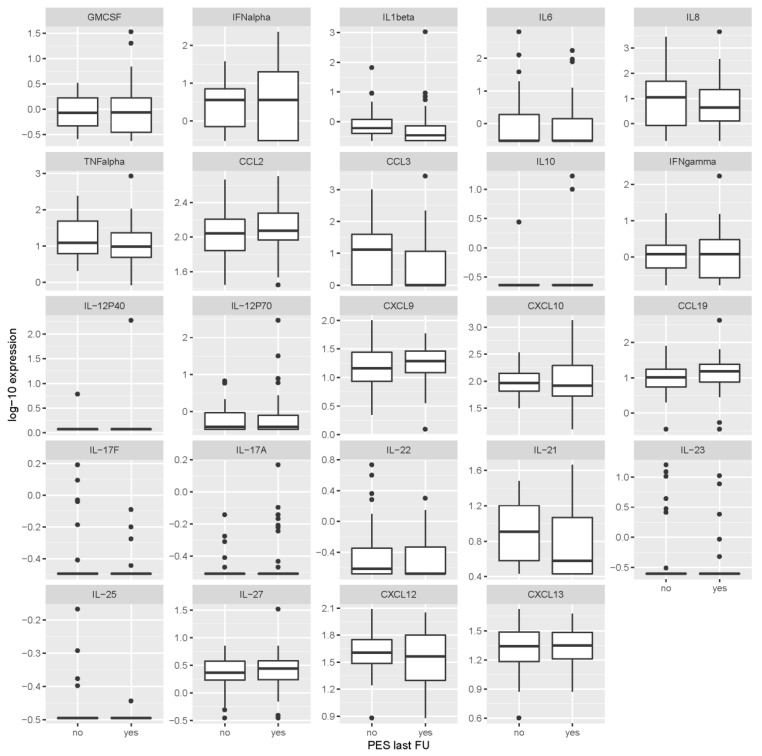
Levels of cytokines/chemokines in serum obtained during the meningoencephalitic phase of tick-borne encephalitis according to the presence or absence of post-encephalytic syndrome at the last follow-up visit (2–7 years after acute illness). Concentrations are in ng/mL for IL-17F, IL-23, IL-25 and IL-27, and in pg/mL for all other inflammatory immune mediators. PES last FU = post-encephalitic syndrome present at last follow-up visit.

**Figure 3:**
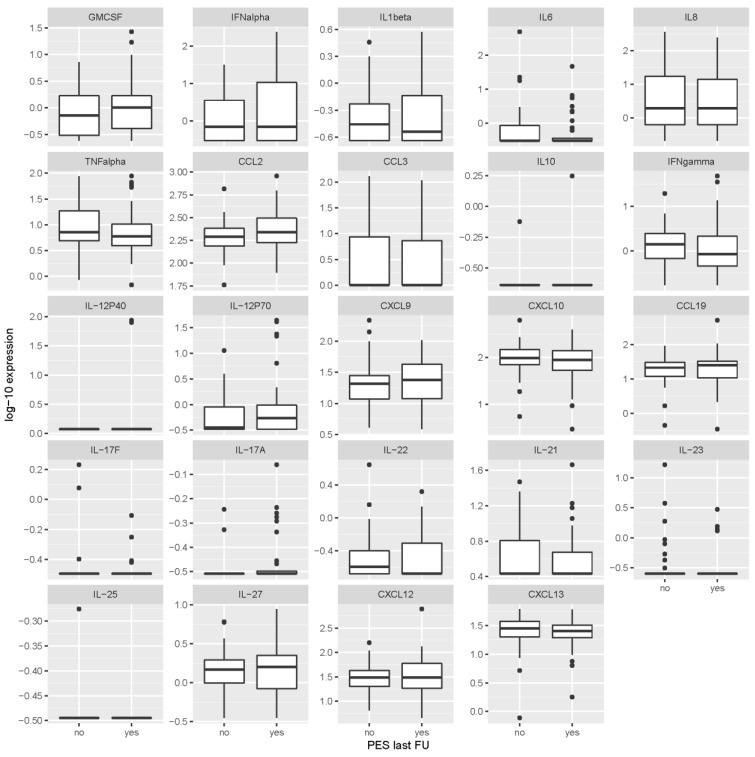
Levels of cytokines/chemokines in serum obtained 2 months after first sampling according to the presence or absence of post-encephalytic syndrome at the last follow-up visit (2–7 years after tick-borne encephalitis). Concentrations are in ng/mL for IL-17F, IL-23, IL-25 and IL-27, and in pg/mL for all other inflammatory immune mediators. PES last FU = post-encephalitic syndrome present at last follow-up visit.

Patients with PES at last visit and those who had a favorable outcome had very similar levels of the individual cytokines/chemokines at the time of acute illness in CSF and serum, and at the 2-month follow-up time point in serum (Figure 1, Figure 2 and Figure 3 and Table 3). None of the mediators was statistically significantly associated with the outcome after correcting the analyses for multiple testing. Thus, based on our data, the cytokines/chemokines levels in CSF or serum within the first 2 months after the onset of TBE did not have predictive value for identifying patients with unfavorable outcome 2–7 years later.

**Figure 4:**
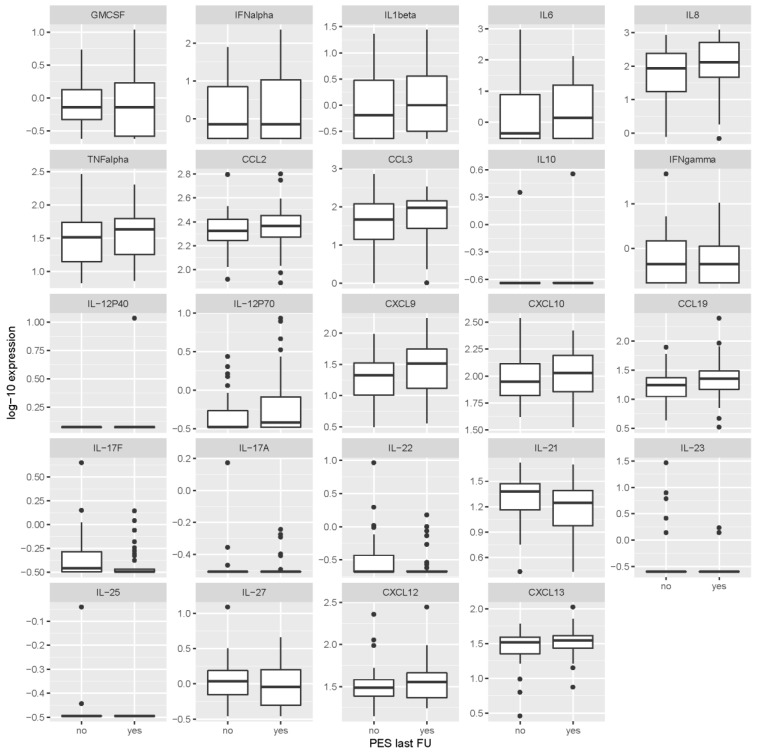
Levels of cytokines/chemokines in serum obtained from patients 2–7 years after tick-borne encephalitis according to the presence or absence of post-encephalytic syndrome at the same time point. Concentrations are in ng/mL for IL-17F, IL-23, IL-25 and IL-27, and in pg/mL for all other inflammatory immune mediators. PES last FU = post-encephalitic syndrome present at last follow-up visit (at the time point when levels of cytokines/chemokines were determined).

However, associations between the levels of inflammatory mediators and unfavorable outcomes were observed at the last visit (Table 3 and Figure 4). At the last visit, 2–7 years after TBE, the levels of Th17 mediators in serum were generally lower in patients with PES. In contrast, the levels of most mediators associated with innate, Th1 or B cell responses were higher for patients with PES at the 2–7 year time point. 

## 4. Discussion

Although most patients recover within weeks following TBE, in some cases objective neurological signs endure and approximately one third of adult patients report persistent symptoms such as fatigue, arthralgias and myalgias, headache, dizziness, sleep disorders, emotional lability, memory and concentration disorders, etc., termed PES. The underlying causes of this syndrome remain unclear. A possible explanation for PES is that these symptoms are due to inappropriate activation of host immune responses following TBEV infection. 

A literature search revealed several reports that have characterized the levels of inflammatory mediators during the meningoencephalitic phase of TBE [12,15,16,17,18,19,20,21,22,23,24,25,26]. Several of these studies demonstrated an association between heightened inflammatory markers and greater disease severity during acute illness [12,19,23,24]. However, only two previous studies evaluated the association of inflammation and disease outcome after TBE. In a study on 44 adult patients with TBE, no correlation between the IFN-γ, TNF-α, IL-10 or IL-6 concentration and the (long-term) outcome was found [19]. In contrast, a study of children (*n* = 22) with TBE, which correlated the levels of 16 cytokines and chemokines and 5 markers of neuronal damage in CSF with long-term outcomes, suggested that the mechanism underlying the central nervous system pathology and TBE sequelae is related to the degree of inflammation in CNS and that high levels of IFN-γ, IL-4, IL-6 and IL-8 in CSF during acute illness might indicate a risk for incomplete recovery in childhood TBE [27]. 

In our current study we comprehensively characterized the inflammatory immune responses in serum and CSF in TBE patients who were followed systematically from acute infection to PES, to better understand the role of the immune responses in the clinical course and outcome of TBE. For this purpose, a cohort of 81 patients who presented with TBE were followed systematically for up to 7 years. Matched serum and CSF samples were collected at the first visit and serum samples were collected at each subsequent visit and correlated with clinical information to identify the type of immune responses related to persistent symptoms associated with PES. Although many cytokines and chemokines may have overlapping functions, we classified them according to the particular immune response they are most commonly associated with to help interpret the results. 

We expected that 2 months after TBE, when all patients were afebrile and the vast majority had improved substantially, their inflammatory immune responses would return to baseline. Indeed, we identified statistically significant differences in the levels of mediators associated with innate, Th1, Th17, and B cell responses between the acute phase and 2 months later. Of interest, statistically significant differences were also observed for the levels of many of these mediators between 2-month time point and 2–7 years after TBE. These results suggest that inflammatory imbalance after TBE is rather long-lasting and would need further assessment. One of the possible explanations for these surprising findings is selection bias due to the larger proportion of patients with PES 2–7 years after TBE (53%) in our group of 81 patients in comparison to the cohort of 420 patients assessed for clinical outcome (33%) [11]. However, differences in serum values of cytokines/chemokines in patients who fulfilled the criteria for PES and those who did not were small and not statistically significant. 

In the present study, the cytokines/chemokines levels in CSF or serum obtained at the time of acute illness as well as serum levels of the inflammatory mediators obtained 2 months after the onset of TBE did not have predictive value for early identification of patients who may have an unfavorable outcome 2–7 years later. However, associations between immune mediators and PES were observed at the last time point (2–7 years after TBE). Of interest, the levels of Th17 mediators in serum tended to be lower for patients with PES at the last time point compared to those whose symptoms resolved, suggesting that more robust Th17 responses may be helpful in disease resolution. In contrast, cytokines/chemokines representing the other immune response groups were higher in patients with PES, supporting the notion that inappropriate immune responses may contribute to adverse clinical outcomes. 

Dysregulated Th17 immunity plays an important role in autoimmune disease, presumably by leading to inappropriate inflammatory immune responses that are associated with tissue pathology, which is implicated in the pathogenesis of several inflammatory and autoimmune diseases including multiple sclerosis and rheumatoid arthritis [28,29,30,31,32,33]. However, Th17 immunity is also important in the control of extracellular pathogens, and may also shape responses to viral infections, both directly and indirectly. The type of Th17 immunity that develops, either pathogenic or protective, is thought to be shaped by the type of stimulus and also by the composition and duration of exposure to the local inflammatory milieu. In patients with Lyme borreliosis, a tick-borne spirochetal infection, Th17 responses appear to be protective during early infection. However, prolonged exposure to high levels of Th17 inflammatory mediators appears to lead to post-treatment complications, including post-Lyme borreliosis symptoms after erythema migrans, and autoimmune phenomena in patients with antibiotic-refractory Lyme arthritis [34]. In viral infections, Th17 responses could cause immunopathology directly by secreting potent pro-inflammatory cytokines and activating local myeloid cells, and indirectly by inhibition of cytotoxic T lymphocyte responses, thereby enhancing viral replication. It is of interest that in the present study the levels of Th17 mediators were not higher but lower or equal in patients having PES in comparison to those with a favorable outcome after TBE, while the levels of several mediators representing the innate or Th1 adaptive responses were typically higher in patients with PES. These findings would suggest that in TBE, robust Th17 responses may play a protective role, whereas prolonged and/or excessive innate or Th1 adaptive responses may be disadvantageous. 

Our study has several limitations. Although the design of the present study enabled insights into the innate or acquired (T and B cells) immune responses and the correlation of these responses with the clinical course and outcome of TBE, CSF was available only at initial visit but not at follow-up visits. Thus, if the inflammatory changes associated with the unfavorable outcome of TBE were occurring predominantly in the central nervous system (CSF) and not in serum, we might miss essential information for the elucidation of the pathogenesis of unfavorable outcome of TBE. For practical and ethical reasons, the access to CSF specimens is limited. In addition, follow-up serum samples in the present study were limited to the time points at 2 months and 2–7 years after TBE. Although we anticipated that inflammatory immune responses would return to baseline 2 months after TBE (these differences were indeed established), we also observed substantial differences in cytokine/chemokine levels between 2 months and 2–7 years after TBE. Assessment of inflammatory mediator levels at additional times, for example at 6 months and/or 12 months after TBE would be needed to ascertain more precisely when the inflammatory responses subsided. Moreover, comparison of serum inflammatory levels in patients with TBE and in healthy control subjects would facilitate the interpretation of findings. Although several statistically significant differences were found comparing serum levels of cytokines/chemokines at different time points, the levels were relatively modest and the interpretation of the biological relevance of the differences remains questionable. Finally, although the number of patients included in the present study (*n* = 81) was substantially higher than in previous reports, this number is still relatively lower than required to overcome the large number of variables in human disease, which could limit the ability to detect significant differences.

In conclusion, our findings demonstrate that infection with TBEV triggers a broad spectrum local (CSF) and systemic (serum) immune response that may take months to years to return to homeostasis. Moreover, dysregulation of these responses years after TBE, as observed with lower Th17 responses but higher innate and Th1 responses, is associated with unfavorable outcomes in patients with PES. These findings provide new insights into the immunopathogenesis of TBE and implicate certain inflammatory immune responses with PES years after the initial infection.

## Figures and Tables

**Table 1:** Basic demographic, clinical, and laboratory data on the acute illness for 81 adult patients with tick-borne encephalitis in whom levels of cytokines and chemokines in serum and cerebrospinal fluid were determined.

Characteristic	Number (%, 95% CI) or Median (IQR)
Male sex	40 (49.4; 38.1–60.7)
Age (years)	56 (43–63)
Males	58 (43–62.5)
Females	55 (45–63)
Underlying illnesses	39 (48.1; 36.9–59.5)
Monophasic course of illness	35 (43.2; 32.2–54.7)
Clinical presentation	
Meningitis	33 (40.7; 30.0–52.2)
Meningoencephalitis	40 (49.4; 38.1–60.7)
Meningoencephalomyelitis	8 (9.9; 4.4–18.5)
Treatment in intensive care unit	7 (8.6; 3.6–17.0)
Duration (days)	5 (3.5–9)
Artificial ventilation: number; duration (days)	2 (28.6; 3.7–71.0); 6 (5–7)
Duration of illness before CSF and first blood sample obtained (days) ^a^	5 (3–6) ^b^
CSF leukocyte count (× 10^6^ cells/L)	76 (37–134)
CSF protein concentration (g/L)	0.70 (0.53–0.91)
Elevated (> 0.45 g/L)	67 (82.7; 72.7–90.2)
Concomitant Lyme neuroborreliosis ^c^	3/78 (3.8; 0.8–10.8)
Positive *B. burgdorferi* sensu lato IgG antibodies ^d^	7/78 (9.0; 3.7–17.6)

^a^ In patients with a biphasic course of illness, this figure was based on the time period from the beginning of the second (meningoencephalitic) phase until hospitalization. ^b^ Data available for 77 patients. ^c^ Diagnosis of concomitant Lyme neuroborreliosis was based on intrathecal synthesis of *Borrelia burgdorferi* sensu lato specific IgG or IgM antibodies or isolation of *B. burgdorferi* sensu lato from CSF. ^d^ The sole indication of borrelial infection. CI, confidence interval; IQR, interquartile range; CSF, cerebrospinal fluid.

**Table 2:** Serum concentrations of cytokines/chemokines obtained in patients with tick-borne encephalitis early in the meningoencephalic phase of the illness, and at follow-up visits 2 months and 2–7 years after tick-borne encephalitis.

Cytokine/Chemokine	Concentrations (pg/mL) Median (IQR)			Ratio of Medians					
	ME Phase of TBE	2 m Later	2–7 y Later	2 m/0 m	*p*^a^ Value	Adjusted *p* ^a^ Value	2–7 y/2 m	*p*^b^ Value	Adjusted *p* ^b^ Value
**Innate**									
GMCSF	0.86 (0.47–1.68)	0.865 (0.31–1.68)	0.72 (0.26–1.68)	1.01	0.956	1.000	0.83	0.423	0.999
IFNα	3.58 (0.30–10.69)	0.71 (0.30–6.16)	0.71 (0.30–10.69)	0.20	<0.001	**0.003**	1.00	0.046	0.511
IL-1β	0.48 (0.24–0.92)	0.32 (0.23–0.69)	0.84 (0.29–3.37)	0.67	0.006	0.080	2.63	<0.001	**<0.001**
IL-6	0.30 (0.30–1.86)	0.30 (0.30–0.76)	0.86 (0.30–14.32)	1.00	0.028	0.332	2.87	<0.001	**<0.001**
IL-8	5.52 (1.05–25.07)	1.91 (0.62–15.06)	108.22 (23.67–314.44)	0.35	0.047	0.502	56.66	<0.001	**<0.001**
TNFα	12.01 (5.38–26.84)	6.43 (4.37–16.39)	39.32 (14.98–60.02)	0.54	<0.001	**0.002**	6.12	<0.001	**<0.001**
MCP1	117.30 (74.27–185.77)	206.25 (158.30–259.33)	223.39 (180.29–269.72)	1.76	<0.001	**<0.001**	1.08	0.069	0.646
MIP1α	1.01 (1.01–30.24)	1.01 (1.01–8.80)	59.21 (17.18–129.04)	1.00	0.014	0.180	58.62	<0.001	**<0.001**
IL-10	0.23 (0.23–0.23)	0.23 (0.23–0.23)	0.23 (0.23–0.23)	1.00	0.789	1.000	1.00	0.789	1.000
**Th1**									
IFNγ	1.1 9(0.45–2.83)	1.05 (0.45–2.43)	0.45 (0.17–1.48)	0.88	0.350	0.999	0.43	<0.001	**<0.001**
IL-12P40	1.18 (1.18–1.18)	1.18 (1.18–1.18)	1.18 (1.18–1.18)	1.00	1.000	1.000	1.00	0.371	0.999
IL-12P70	0.38 (0.33–0.92)	0.38 (0.33–0.92)	0.33 (0.33–0.72)	1.00	0.647	1.000	0.87	0.011	0.153
CXCL9	17.42 (10.22–28.51)	22.21 (11.67–40.55)	24.01 (12.11–40.78)	1.28	0.002	**0.023**	1.08	0.771	1.000
CXCL10	88.34 (56.41–171.02)	91.1 9(59.58–147.12)	101.97 (69.51–147.12)	1.032	0.624	1.000	1.12	0.586	1.000
CCL19	11.82 (6.21–21.16)	24.08 (11.42–31.48)	20.07 (11.98–28.47)	2.04	<0.001	**<0.001**	0.83	0.051	0.543
**Th17**									
IL-17F ^c^	0.32 (0.32–0.32)	0.32 (0.32–0.32)	0.32 (0.32–0.47)	1.00	0.289	0.997	1.00	<0.001^d^	**0.002**
IL-17A	0.31 (0.31–0.31)	0.31 (0.31–0.31)	0.31 (0.31–0.31)	1.00	0.018	0.231	1.00	0.364	0.999
IL-22	0.21 (0.21–0.45)	0.22 (0.21–0.47)	0.21 (0.21–0.34)	1.02	0.319	0.999	0.98	0.058	0.588
IL-21	5.70 (2.71–14.11)	2.71 (2.71–4.75)	20.31 (11.35–27.41)	0.48	<0.001	**<0.001**	7.49	<0.001	**<0.001**
IL-23 ^c^	0.25 (0.25–0.25)	0.25 (0.25–0.25)	0.25 (0.25–0.25)	1.00	0.224	0.988	1.00	0.182	0.961
IL-25 ^c^	0.32 (0.32–0.32)	0.32 (0.32–0.32)	0.32 (0.32–0.32)	1.00	0.281	0.997	1.00	1.000	1.000
IL-27 ^c^	2.63 (1.70–3.86)	1.52 (0.90–2.12)	0.94 (0.59–1.54)	0.58	<0.001	**<0.001**	0.62	<0.001	**<0.001**
**B cell**									
CXCL12	22.40 (16.30–30.84)	26.24 (19.40–36.08)	34.36 (25.30–40.26)	0.78	0.003	**0.040**	1.31	0.169	0.935
CXCL13	39.26 (24.44–61.32)	30.74 (18.98–52.88)	33.59 (23.29–41.51)	1.17	0.075	0.683	1.09	<0.001	**0.005**

The Wilcoxon signed rank test with continuity correction was used for comparisons of individual cytokines/chemokines; univariate *p* values were adjusted for multiple comparisons (adjusted *p*) with a multivariate permutation procedure. *p* values for significantly different concentrations are shown in bold. ^a^ Comparison of findings during acute illness and 2 months later. ^b^ Comparison of findings obtained at visit 2 months and 2–7 years after TBE. ^c^ Concentrations are given in ng/mL. ^d^ Medians and IQRs are the same but the other data (in the 1st and 4th quartiles) are not. IQR, interquartile range; ME, meningoencephalitic; TBE, tick-borne encephalitis; m, month, y, year.

**Table 3:** Correlation of cytokine and chemokine levels obtained at the time of acute illness, 2 months later, and 2–7 years later with the outcome of tick-borne encephalitis 2–7 years after acute illness.

Cytokine/Chemokine	Unfavorable Outcome * of Tick-Borne Encephalitis 2–7 Years after Acute Illness											
	CSF, Time 0			Serum, Time 0			Serum, Time 2 Months			Serum, Time 2–7 Years		
	Median Ratio ^a^ (PES/Others)	*p* Value ^b^	Adjusted *p* Value ^c^	Median Ratio ^a^ (PES/Others)	*p* Value ^b^	Adjusted *p* Value ^c^	Median Ratio ^a^ (PES/Others)	*p* Value ^b^	Adjusted *p* Value ^c^	Median Ratio ^a^ (PES/Others)	*p* Value ^b^	Adjusted *p* Value ^c^
**Innate**												
GMCSF	0.84	0.728	1	0.99	0.742	1	1.40	0.378	1	1	0.943	1
IFNα	1	0.573	1	1	0.627	1	1	0.598	1	1	0.914	1
IL-1β	0.93	0.996	1	0.57	0.004	0.215	0.83	0.921	1	1.54	0.530	1
IL-6	1.48	0.206	1	1	0.598	1	1	0.411	1	3.10	0.332	1
IL-8	1.08	0.891	1	0.40	0.538	1	1	0.944	1	1.52	0.191	1
TNFα	1.11	0.974	1	0.78	0.085	0.998	0.83	0.396	1	1.31	0.590	1
MCP1	1.01	0.914	1	1.07	0.342	1	1.12	0.234	1	1.10	0.348	1
MIP1α	0.56	0.021	0.769	0.08	0.069	0.995	1	0.840	1	2.02	0.235	1
IL-10	1	0.910	1	1	0.623	1	1	0.969	1	1	0.958	1
**Th1**												
IFNγ	1.37	0.274	1	1	0.842	1	0.61	0.425	1	1	0.598	1
IL-12P40	0.82	0.247	1	1	0.958	1	1	0.149	1	1	0.360	1
IL-12P70	1.07	0.636	1	1	0.938	1	1.52	0.117	1	1.15	0.385	1
CXCL9	1.37	0.349	1	1.34	0.302	1	1.15	0.581	1	1.55	0.083	0.998
CXCL10	1.01	0.573	1	0.89	0.913	1	0.90	0.312	1	1.20	0.424	1
CCL19	1.65	0.061	0.990	1.48	0.130	1	1.18	0.638	1	1.30	0.026	0.837
**Th17**												
IL-17F	1	NA	NA	1	0.316	1	1	0.709	1	0.91	0.079	0.998
IL-17A	1	NA	NA	1	0.298	1	1	0.021	0.767	1	0.405	1
IL-22	1	0.135	1	0.86	0.193	1	0.82	0.790	1	1	0.054	0.982
IL-21	1	0.931	1	0.47	0.034	0.915	1	0.573	1	0.74	0.031	0.879
IL-23	1	NA	NA	1	0.222	1	1	0.734	1	1	0.159	1
IL-25	1	NA	NA	1	0.120	1	1	0.344	1	1	0.135	1
IL-27	1.14	0.769	1	1.19	0.596	1	1.09	0.657	1	0.83	0.525	1
**B cell**												
CXCL12	0.44	0.513	1	0.90	0.364	1	1.00	0.863	1	1.17	0.339	1
CXCL13	1.12	0.691	1	1.02	0.744	1	0.91	0.341	1	1.07	0.230	1

* Unfavorable outcome was defined by the presence of post-encephalitic syndrome. ^a^ Mann-Whitney tests were used to compare the levels of the examined cytokines/chemokines at first visit and follow-up times in the group of patients with and without post-encephalitic syndrome at the last follow-up visit. We reported the fold change of the medians (defined as the ratio of medians observed in the group of patients with post-encephalitic syndrome and in the group without post-encephalitic syndrome at the last follow-up visit; values > 1 indicate greater median concentrations in the group with post-encephalitic syndrome. ^b^ *p* values from the Mann-Whitney tests. ^c^ *p* values from the Mann-Whitney tests adjusted for multiple comparisons. CSF, cerebrospinal fluid; NA, not available (because no variability in concentrations was present).

## Supplementary Materials

The following are available online at https://www.mdpi.com/2076-2607/7/11/514/s1. Figure S1: Concentrations of inflammatory immune markers in serum of patients with tick-borne encephalitis at the time of acute illness (up to 14 days from the beginning of meningoencephalitic phase of the disease), 2 months later, and 2–7 years after the onset of the disease. Concentrations are in ng/mL for IL-17F, IL-23, IL-25 and IL-27, and in pg/mL for all other inflammatory immune mediators. Last FU = last follow-up visit (2–7 years after the onset of tick-borne encephalitis).
