# Supplementary material for: Inflammatory Immune Responses in Patients with Tick-Borne Encephalitis: Dynamics and Association with the Outcome of the Disease
Source: Microorganisms. 2019 Oct 31;7(11):514. doi: 10.3390/microorganisms7110514 (PMC6920956; doi:10.3390/microorganisms7110514)
Supplement: Supplementary file 1 [file microorganisms-07-00514-s001.pdf]

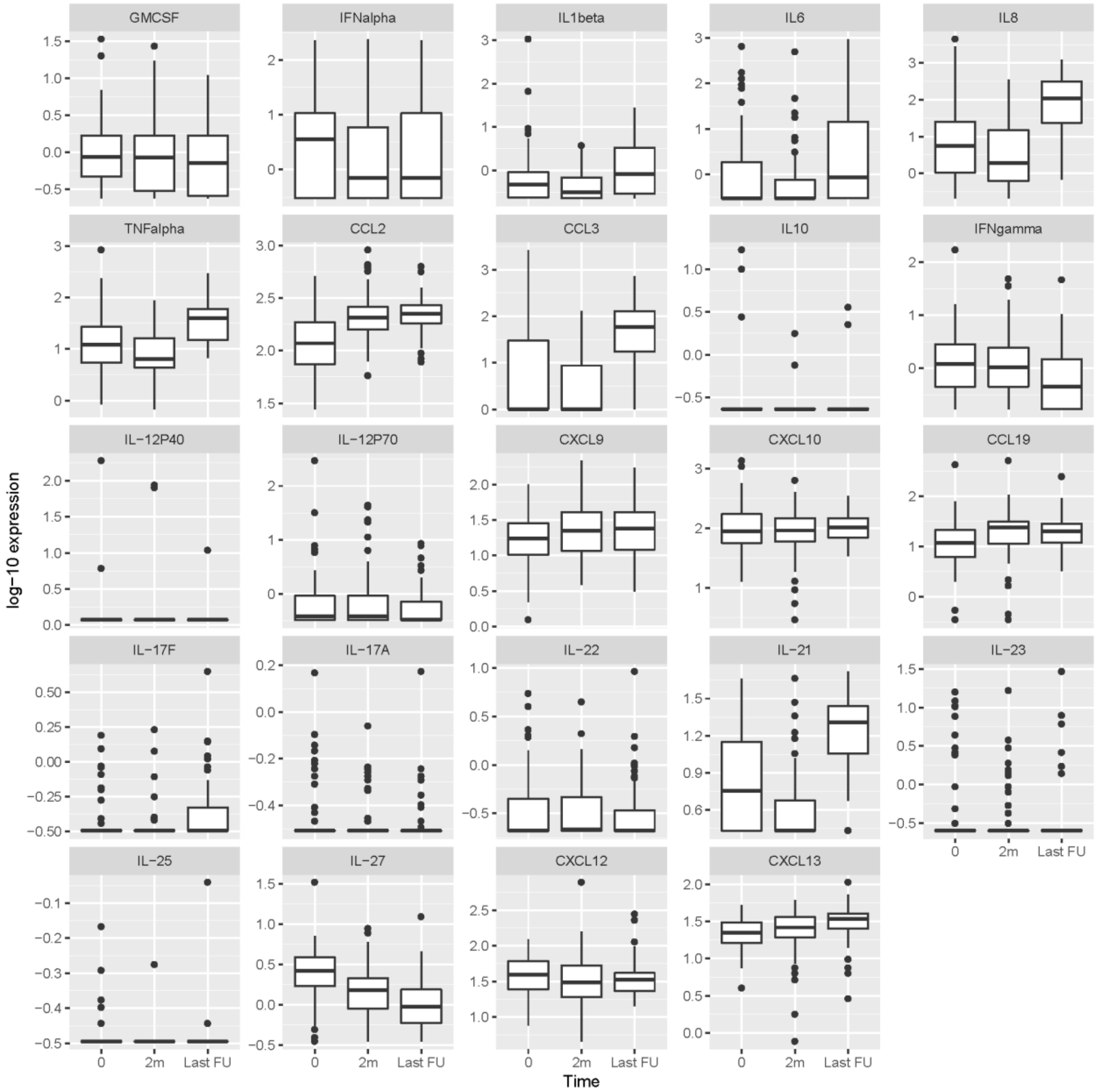

Supplementary Figure 1. Concentrations of inflammatory immune markers in serum of patients with tick-borne encephalitis at the time of acute illness (up to 14 days from the beginning of meningoencephalitic phase of the disease), 2 months later, and 2–7 years after the onset of the disease. Concentrations are in ng/mL for IL-17F, IL-23, IL-25 and IL-27, and in pg/mL for all other inflammatory immune mediators. Last FU = last follow-up visit (2–7 years after the onset of tick-borne encephalitis)
